# Application of Hepatocyte Growth Factor for Acute Spinal Cord Injury: The Road from Basic Studies to Human Treatment

**DOI:** 10.3390/ijms20051054

**Published:** 2019-02-28

**Authors:** Kazuya Kitamura, Narihito Nagoshi, Osahiko Tsuji, Morio Matsumoto, Hideyuki Okano, Masaya Nakamura

**Affiliations:** 1 Department of Orthopedic Surgery, Saiseikai Yokohamashi Tobu Hospital, Kanagawa 230-0012, Japan; kazuya@mre.biglobe.ne.jp; 2 Department of Orthopedic Surgery, Keio University School of Medicine, Tokyo 160-8582, Japan; nagoshi@2002.jukuin.keio.ac.jp (N.N.); osahiko@2003.jukuin.keio.ac.jp (O.T.); morio@a5.keio.jp (M.M.); 3 Department of Physiology, Keio University School of Medicine, Tokyo 160-8582, Japan

**Keywords:** spinal cord injury, hepatocyte growth factor, recombinant human hepatocyte growth factor, clinical trial

## Abstract

Hepatocyte growth factor (HGF) was first identified as a potent mitogen for mature hepatocytes, and has also gained attention as a strong neurotrophic factor in the central nervous system. We found that during the acute phase of spinal cord injury (SCI) in rats, c-Met, the specific receptor for HGF, increases sharply, while the endogenous HGF up-regulation is relatively weak. Introducing exogenous HGF into the spinal cord by injecting an HGF-expressing viral vector significantly increased the neuron and oligodendrocyte survival, angiogenesis, and axonal regeneration, to reduce the area of damage and to promote functional recovery in rats after SCI. Other recent studies in rodents have shown that exogenously administered HGF during the acute phase of SCI reduces astrocyte activation to decrease glial scar formation, and exerts anti-inflammatory effects to reduce leukocyte infiltration. We also reported that the intrathecal infusion of recombinant human HGF (intrathecal rhHGF) improves neurological hand function after cervical contusive SCI in the common marmoset, a non-human primate. Based on these collective results, we conducted a phase I/II clinical trial of intrathecal rhHGF for patients with acute cervical SCI who showed a modified Frankel grade of A/B1/B2 72 h after injury onset, from June 2014 to May 2018.

## 1. Introduction

Spinal cord injury (SCI) is a devastating impairment, with an estimated overall annual incidence of 15–53 cases per million [1,2]. Most cases of SCI are related to physical activity (motor vehicle accidents, falls, violent crimes, and sports injuries), and affect individuals in their second and third decades of life [3,4]. However, the incidence of SCI in people over 60 has increased in recent years [1,5,6]. Notably, the second highest incidence of SCI occurs in people over 50, and pre-existing spondylosis is associated with SCI, which in this case can result from a relatively low-energy trauma [3,4]. Given that the population is rapidly aging worldwide, the establishment of a comprehensive treatment strategy for SCI patients of all generations is desired.

SCI begins with mechanical compression to the spinal cord, followed by secondary damage, which includes spinal cord ischemia, hemorrhage, cellular excitotoxicity, hyper-permeability, ionic dysregulation, and free-radical-mediated peroxidation [7,8]. Although many therapeutic experiments have been conducted using neurotrophic factors to reduce the secondary damage and to enhance axonal regrowth, an effective neuroprotective strategy for humans has not been established. To date, massive methylprednisolone sodium succinate (MPSS) administration [9] is the only neuroprotective therapy approved for acute SCI, but its efficacy and safety remain controversial [10,11,12,13]. Hepatocyte growth factor (HGF) was first cloned as a mitogen for mature hepatocytes [14,15,16], and the specific receptor of HGF was identified as the c-Met proto-oncogene product, a transmembrane receptor with a tyrosine kinase domain in its intracellular domain [17]. All of the diverse biological activities of HGF, including roles in cell survival, proliferation, and migration, are induced by c-Met activation [18]. In addition to its roles in controlling development and tissue homeostasis under normal physiological conditions, the HGF–c-Met system has gained attention for its ability to exert regenerative effects, including angiogenic activity, after tissue injury in many epithelial organs [19]. In the central nervous system, the HGF–c-Met system has been shown to control neuronal development. Studies in rodent models have revealed that HGF administration promotes angiogenic activity, prevents disruption of the blood–brain barrier [20,21,22], and promotes the survival of neurons both after cerebral ischemia [22,23,24,25,26,27] and in a transgenic amyotrophic lateral sclerosis (ALS) model [28,29]. A treatment strategy using HGF for central nervous system disorders in humans was first applied for ALS. A phase I study of intrathecally administered recombinant human HGF (rhHGF) for ALS was conducted from 2011 to 2015 at Tohoku University, Japan [30], and a phase II study began in May 2016.

In 2007, we reported that a sharp increase in c-Met expression occurs in the injured spinal cord of a rat 1 day after SCI, while the upregulation of endogenous HGF is relatively weak, with a peak 2 weeks after the injury. Introduction of exogenous HGF into the spinal cord by injecting an HGF-expressing herpes simplex virus (HSV) vector significantly increased the survival of neurons and oligodendrocytes, angiogenesis, and axonal regeneration, to reduce the area of damage and promote motor function of the hind limbs after SCI in rats [31]. Since that first report, we have developed this strategy from rodents to primates, and from gene therapy to clinically available rhHGF, which is intrathecally administered. This therapy was tested in a phase I/II clinical trial for acute cervical SCI from June 2014 to May 2018 in Japan (clinicaltrials.gov, registration number NCT02193334). Here we present our research journey from the bench to clinical trial, in which we seek to establish a new effective treatment for acute SCI using rhHGF.

### 1.1. Time Course of the Endogenous Hepatocyte Growth Factor–c-Met System after Spinal Cord Injury in Rats: Endogenous Hepatocyte Growth Factor Upregulation is Weak in the Spinal Cord during the Acute Injury Phase

The HGF–c-Met system is known to mediate inflammatory responses to tissue injury. In animal injury models of the liver [32,33,34], lung [35], or kidney [32,36], the level of HGF activity significantly increased in the damaged organs, with a peak within 1 day after the injury (Table 1). Similarly, the plasma HGF level also increased within 1 day after the injury, suggesting that HGF can be delivered to the damaged organ from distal intact organs by an endocrine mechanism, in addition to being produced in the damaged organs themselves (Table 1) [32]. However, in the central nervous system, unlike in the liver or kidney, the upregulation of HGF mRNA at an injured brain region was delayed, peaking 14 days after injury onset in mice [37]. 

Given this background, we first examined the endogenous HGF–c-Met system activity over time in the adult rat spinal cord after contusive damage. The HGF mRNA expression slowly increased, with a peak 2 weeks after the damage, while the c-Met mRNA expression increased sharply within a day. Consistent with the slow increase in HGF mRNA expression, the HGF protein level in the damaged spinal cord showed a gradual increase, with a peak around 4 weeks after the injury. Furthermore, the HGF protein level in the plasma did not show any significant increase after the damage. These findings suggested that a spinal cord with contusive damage cannot upregulate endogenous HGF expression by itself, in contrast to the marked upregulation of c-Met expression after the damage—nor can the HGF protein be delivered from distal intact organs by an endocrine mechanism, unlike in other damaged epithelial organs (Table 1) [31]. Based on these primary findings, we hypothesized that the administration of exogenous HGF into the damaged spinal cord immediately after the onset of injury would exert therapeutic effects for tissue sparing or regeneration.

### 1.2. Introducing Exogenous Hepatocyte Growth Factor into the Spinal Cord using the Herpes Simplex Virus-1 Vector Exerted Multiple Therapeutic Effects to Promote Functional Recovery in a Rat Thoracic Spinal Cord Injury Model: Neuroprotection and Promotion of Angiogenic Activity and Axonal Regrowth

To administer exogenous HGF into the spinal cord, we first performed gene therapy in rats, in which an HGF-expressing herpes simplex virus-1 (HSV-1) vector was injected directly into the spinal cord. Three days after HSV-1 vector injection at the tenth thoracic vertebral level (T10), the exogenous HGF was expressed in the spinal cord at 3–4 times the endogenous HGF level, and contusive injury was induced at the same level to examine HGF’s therapeutic effects on the damaged spinal cord (Table 2) [31]. The exogenous HGF significantly decreased the damaged area and promoted motor function of the hindlimbs 6 weeks after the onset of injury. The cavity area of the injured spinal cord was significantly reduced, and the rim of myelinated white matter was significantly preserved. To investigate this tissue-sparing effect, immunohistological and immunoblotting analyses were performed. These analyses showed that c-Met-immunoreactivity (c-Met-IR) was present in the neurons, oligodendrocytes, and reactive astrocytes after SCI, indicating that HGF would have biological activity in all three of these cell lineages in the damaged spinal cord [31]. 

HGF has been recognized as a neuroprotective factor for a variety of neurons [38,39,40]. In an experimental animal model, the administration of HGF showed neuroprotective effects on adult motor neurons after axotomy of the hypoglossal nerve, by maintaining the choline acetyltransferase (ChAT) activity in the nucleus [41]. HGF application was also reported to promote the survival of several types of neurons in the brain after cerebral ischemia [22,23,24,27,42] and of motor neurons in the spinal cord in transgenic ALS model mice and rats, by attenuating the levels of caspase-1 and inducible nitric oxide synthase in motor neurons, as well as by maintaining the level of the glial-specific glutamate transporter in reactive astrocytes [28,29]. Immunoblotting analyses of spinal cord lysates 3 days after SCI revealed that HGF significantly attenuated the levels of cleaved caspase-3 in the damaged spinal cord. Immunohistological analyses have also shown that HGF reduces the numbers of cleaved caspase-3-positive motor neurons and oligodendrocytes, indicating that HGF reduces their apoptosis, thereby promoting their survival. Accordingly, significantly greater numbers of surviving motor neurons in the ventral horns have been observed in an HGF-treated than in the control group 6 weeks after SCI [31]. 

HGF has also been shown to enhance angiogenic activity; c-Met is expressed in endothelial cells and vascular smooth muscle cells (VSMCs), and HGF simultaneously promotes the migration of endothelial cells and VSMCs [43,44], indicating that blood vessels may undergo maturation without enhancing their permeability or causing inflammatory cells to be released [45]. In a cerebral infarction animal model, HGF exerted neuroprotective effects and promoted angiogenesis without exacerbating the cerebral edema, and thus reduced the ischemic damage [22]. Even in the pathology of contusive SCI, studies indicated that the microvasculature in the damaged spinal cord, especially within 1 week after injury onset, plays a key role in maintaining the ability of the spinal cord to undergo endogenous repair, by supplying nutritional and mechanical support for axonal regeneration [46,47,48,49], and several strategies to enhance angiogenic activity in the damaged spinal cord were shown to contribute to improved motor function [50,51]. Our immunohistological analysis revealed that HGF exogenously administered into the damaged spinal cord increased the number of newly formed vessels [49] 1 week after injury onset, confirming that HGF enhanced the angiogenic activity [31], as seen in the cerebral infarction animal models. Several groups have reported that HGF exerts chemoattractive and growth-promoting effects on the axons of motor neurons [38,52,53] and cortical neurons [54]. Furthermore, overexpressing HGF during the chronic stage of cerebral infarction promotes neurite outgrowth and synapse formation, contributing to improvements in learning and memory [25]. In our study, HGF promoted the regrowth of raphe-spinal 5HT-positive fibers, which are reported to be involved in motor functional recovery in rats, 6 weeks after SCI, [55,56,57]. These fibers expressed GAP-43, a marker of axonal regeneration [58,59,60], and c-Met, suggesting that HGF might have promoted motor functional recovery by directly enhancing the regrowth of these fibers [31].

### 1.3. Other Recent Basic Studies on Hepatocyte Growth Factor Treatment for Spinal Cord Injury

#### 1.3.1. Reduction of Astrocyte Activation and Chondroitin Sulfate Proteoglycan Deposition

Glial scars, which form at the injury site after SCI by an astrocytic response, contain extracellular matrix molecules like chondroitin sulfate proteoglycans (CSPGs), which inhibit axonal growth [61,62]. Thus, overcoming this inhibitory environment is an important focus in the search for therapeutic interventions to enhance axonal growth and promote functional recovery. Jeong et al. reported that HGF prevents the expression of specific CSPG species by blocking the secretion of TGFβ1 and β2 from reactive astrocytes in vitro. They further found that transplanting HGF-overexpressing mesenchymal stem cells (HGF-MSCs) into hemisectional cervical spinal cord lesions significantly decreased the TGFβ isoform levels, reduced astrocyte activation, and decreased CSPG deposition around the lesion site (Table 2). Finally, they confirmed that HGF-MSC transplantation increased axonal growth beyond the glial scars, and promoted functional recovery of the forepaw compared to controls [63]. 

#### 1.3.2. Anti-Inflammatory Effect and Reduction in Leukocyte Infiltration

HGF is reported to exert anti-inflammatory effects by disrupting nuclear factor-kappa B (NF-κB) signaling and the subsequent expression of NF-κB-dependent pro-inflammatory mediators [64,65]. Yamane et al. investigated the efficacy of an engineered HGF fused with a collagen-binding domain (CBD-HGF), which was detected in the mouse spinal cord for 7 days after its intrathecal injection, while unmodified HGF almost disappeared after 1 day. The authors revealed that a single intrathecal injection of CBD-HGF immediately after thoracic compressive SCI in mice significantly prevented the infiltration of leukocytes, such as neutrophils and macrophages, compared to a single intrathecal injection of unmodified HGF, by attenuating cytokine and chemokine levels at the injury site (Table 2) [66]. This anti-inflammatory effect reduced the glial scar formation and promoted axonal regrowth and functional recovery. This study was noteworthy, not only because it reported the anti-inflammatory effect of HGF on the injured spinal cord, but also because it revealed that HGF concentration and distribution in the spinal cord need to be maintained during the acute phase of SCI to exert therapeutic effects. 

#### 1.3.3. Increasing the Survival, Neuronal Differentiation, and Synapse Formation of Grafted Neural Stem Cells to Promote Functional Recovery

Despite the increased incidence of SCI in people over 60 in recent years [5,6], the pathophysiology of SCI and the efficacy of cell transplantation therapy in older animals have not been fully understood. Therefore, our group [67] investigated the efficacy of neural stem cell (NSC) transplantation in aged mice with SCI. Aged mice actually had a better capacity for the survival and neuronal differentiation of grafted NSCs than young mice, and the grafted NSC-derived neurons formed synapses with descending corticospinal fibers more efficiently in the aged mice than in the young ones, thereby promoting functional recovery of the hindlimbs. Based on these surprising results, microarray analysis of damaged spinal cord tissue 9 days after SCI was performed to investigate the neurotrophic factors involved. This analysis revealed that HGF was the most highly expressed among various neurotrophic factors in the aged mice compared to the young ones (Table 2). Notably, inhibiting HGF with neutralizing antibodies significantly decreased the survival, neuronal differentiation, and synapse formation of the grafted NSCs, along with the functional recovery of the mice. In addition, the HGF-inhibiting, antibody-treated mice exhibited more severe spinal cord atrophy and demyelination than the control mice. This study was the first to report that HGF promotes the neuronal differentiation of grafted NSCs in the injured spinal cord, consistent with previous in vitro results [68,69]. Taken together, HGF was identified as an important factor for the enhanced survival, differentiation, and synapse formation of grafted NSCs, which promoted functional recovery. Importantly, this finding implies that a combination therapy involving HGF administration during SCI’s acute phase followed by NSC transplantation may be a promising therapeutic strategy for patients of any age, including older patients.

### 1.4. Summary of the Therapeutic Mechanisms of Hepatocyte Growth Factor in Rodents

**Figure 1:**
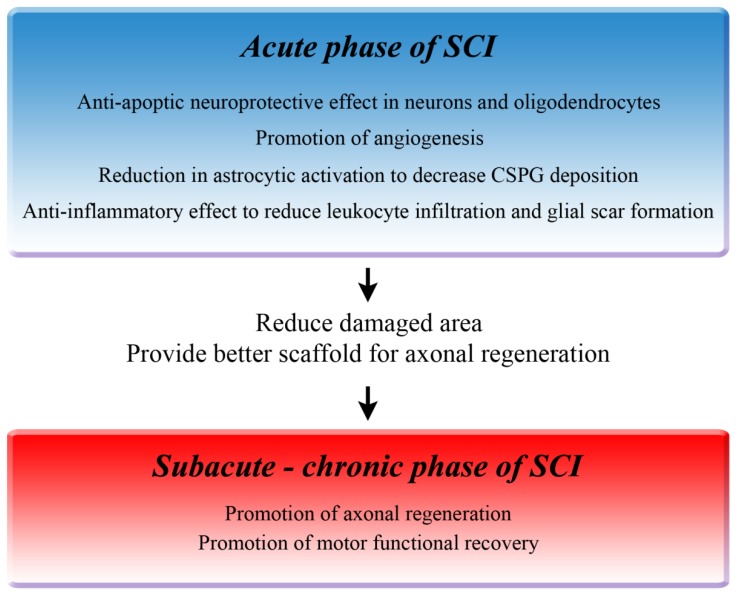
Therapeutic mechanisms of hepatocyte growth factor (HGF). Preclinical studies using rodent spinal cord injury (SCI) models revealed multiple therapeutic effects of HGF on the injured spinal cord that reduce the damaged area and promote functional recovery.

As shown in Figure 1, during the acute phase of SCI, HGF promotes the survival of neurons and oligodendrocytes by preventing apoptosis, and enhances angiogenic activity around the lesion site [31]. Moreover, HGF reduces astrocyte activation to decrease CSPG deposition [63] and reduces infiltrating leukocytes, thereby contributing to a reduction in glial scar formation [66]. These multiple effects reduce the damaged area, preserve the rim of myelinated white matter, and provide a better scaffold for axonal regrowth. Furthermore, HGF directly enhances the regrowth of 5HT-positive fibers, which probably contributes to the promotion of motor functional recovery of the hind limbs during the chronic phase of SCI [31]. In cell-based therapeutic interventions, HGF promotes the survival, neuronal differentiation, and synapse formation of the grafted neural stem cells in the damaged spinal cord, and these combined effects contribute to the better functional recovery [67].

## 2. Steps Toward Starting a Clinical Trial

### 2.1. Translation from Gene Therapy to the Intrathecal Administration of Recombinant Human Hepatocyte Growth Factor, and from Rat Thoracic Spinal Cord Injury to Primate Cervical Spinal Cord Injury 

To develop this HGF strategy into a clinical treatment for SCI patients, we performed a series of in vivo studies to confirm the efficacy of the intrathecal infusion of rhHGF, starting from the acute phase of SCI, which could be applied to human treatment.

#### 2.1.1. Advantages of Using a Cervical SCI Model in Marmosets for Preclinical Trials

First, we initiated the intrathecal administration of rhHGF immediately after contusive cervical SCI in adult marmosets, a non-human primate (common marmoset: *Callithrix Jacchus*). Although cervical SCI, which causes an impairment in upper-limb motor function (including finger dexterity), is more frequent than thoracic SCI in humans [3], it is difficult to evaluate finger dexterity or hand function in rodents. Thus, to better predict the therapeutic effects for human cervical SCI, a preclinical trial using a cervical SCI model in non-human primates would enable us to evaluate the recovery of upper-limb motor function, including that of the hand. In marmosets, the corticospinal tract (CST) runs in the lateral column like in humans, but projects to the dorsal horn and the intermediate zone of the gray matter without a visible, direct cortico-motoneuronal (CM) connection like in rodents, indicating that the marmoset may represent an intermediate animal between rodents and macaque monkeys or humans, with respect to neuroanatomical evolution [70,72]. Marmosets cannot perform dexterous finger actions, such as pinching something between the index finger and the thumb, like macaque monkeys can, but marmosets show more highly developed reach and grasp performance than rodents [70]. In these experiments, the spinal motor neurons that innervate the muscles in the marmoset forelimbs were first retrogradely labeled by injecting cholera toxin B subunit (CTB) into the muscles. The motor neurons for wrist and finger extension were found to be located mainly in the cervical (C)4-C7 segments, and those for wrist flexion were located in the C6-T1 segments. Therefore, we induced a contusive SCI at C5. Nearly all of the wrist extensor and flexor motor neurons were caudal to the lesion epicenter, suggesting that this injury would be useful for evaluating hand functions. Next, to assess the hand performance after cervical SCI in these animals, we designed an open field rating scale [70], in which the position and placement of the hands during walking were recorded. The positions were below the shoulder, between the shoulder and head, and above the head; and the placements were dorsal, ulnar but not pronated, pronated but not palmar, and palmar. The reach-and-grasp performance was also examined to assess shoulder flexion, forearm pronation, wrist extension, and finger flexion and extension.

#### 2.1.2. Intrathecal Recombinant Human Hepatocyte Growth Factor Promotes Neurological Hand Function and Reduces the Damaged Area in a Non-Human Primate after Spinal Cord Injury

In adult female marmosets, a laminectomy was performed at the C5 level. The dura mater was then exposed, and contusive SCI was elicited at the C5 level by dropping a 20-g weight from a 50-mm height onto the dura by a modified-New York University (NYU) impactor [73,74,75]. Immediately afterwards, a laminectomy at the C7 level was performed, and from this level an intrathecal catheter was inserted. An osmotic mini pump infused a total of 400 µg of rhHGF or phosphate buffered saline (control group) for 4 weeks; the dosage was based on previous experiments, in which rhHGF was intrathecally administered into ALS model rats [29], which weigh about the same as marmosets. KP-100IT, a pharmaceutical preparation containing rhHGF as the active ingredient and manufactured under cGMP compliant conditions, was provided by Kringle Pharma, Inc. (Osaka, Japan).

The results using our original open field scale showed that the rhHGF-treated and control groups had significant differences in neurological hand function. One day after the injury, all of the animals exhibited severe quadriplegia, which gradually improved, with a plateau beginning approximately 8 weeks after SCI. At 12 weeks after SCI, the score for the upper limbs (pre-injury score = 20) improved to 15.9 ± 0.8 in the rhHGF group and to 7.8 ± 1.8 in the control group. The scores for the two groups were significantly different at most of the time points on or after 14 days post-SCI. The animals in the rhHGF group also showed better grasping performance than the control group, and exhibited palmar hand placement when walking, with their wrist joints extended and forearms pronated, whereas animals in the control group dragged the dorsal surface of their hands on the floor when walking, with their wrists dropped [70].

To examine the tissue-sparing effects of intrathecal rhHGF, we stained axial sections of injured spinal cords with Luxol fast blue (LFB) 12 weeks after SCI. In contrast to the control group, the rhHGF group exhibited a rim of spared myelinated white matter, even at the lesion epicenter, similar to our previous rat study [31]. Moreover, stereological quantification of the calmodulin-dependent kinase II α(CaMK II α)-positive region in the lateral column, which represents the CST in the marmoset spinal cord [73,75,76], showed significant differences between the rhHGF-treated and control groups at the segments that were caudal to the lesion site. These pathological findings suggest that intrathecal rhHGF can exert tissue-sparing effects on a cervical spinal cord with contusive injuries in non-human primates. To the best of our knowledge, this study provides the first evidence for the therapeutic efficacy of a neuroprotective drug for contusive SCI in a non-human primate [70].

#### 2.1.3. Therapeutic Efficacy of Intrathecally Administered Recombinant Human Hepatocyte Growth Factor in a Severe Cervical Spinal Cord Injury Marmoset Model 

As a final step before starting a clinical trial, we also confirmed the efficacy of intrathecal rhHGF in promoting upper limb motor function in a clinically relevant, more severe cervical SCI model in the common marmoset. Contusive SCI was induced at the C5 level, as described above. All eight marmosets included in this final test showed flaccid fingers, without any active flexion or extension; slight movement of the shoulder and elbow, which showed less than half the normal range of motion; and no ability of the upper limbs to support weight (0 points in the original open field rating scale) until 4 days after SCI. The initial step of the motor functional recovery of the upper limbs (the first point in the original open field rating scale) was characterized by the elevation of the marmoset’s hands up to head height, with shoulder and elbow flexion. Three animals in the control group showed almost no recovery, even after 4 weeks, with all the key muscles of the upper limbs remaining useless (0.5 ± 0.3 points in the original open field rating scale). In contrast, the intrathecal infusion of 400 µg of rhHGF for 4 weeks starting right after SCI led to significantly improved functional recovery of the upper limbs (4.0 ± 1.8) compared to the control group. All five marmosets in the rhHGF group recovered to raise their hands up to their head height and gained more than 1 point, suggesting that at least one key muscle became useful. We believe that this model was ideal for a preclinical trial, because it is a contusive cervical SCI model in a non-human primate in which the control animals show no recovery; thus, the effect of the therapeutic intervention could be clearly assessed. This final test showed the promising result that intrathecal rhHGF, when started during the acute phase of SCI, could induce a clinically important improvement in upper-limb motor function, even in a severe cervical SCI primate model [71].

### 2.2. Therapeutic Time Window and Minimal Effective Dose of Intrathecal Recombinant Human Hepatocyte Growth Factor

#### 2.2.1. Delayed Intrathecal Recombinant Human Hepatocyte Growth Factor startinga 4 Days after SCI Improved the Functional Recovery after Spinal Cord Injury in Rats

To examine the dose and timing of delayed intrathecal rhHGF treatment, a contusive SCI was induced in adult rats at the tenth thoracic vertebrate level (T10), as in our previous study [31]. Immediately after this injury, a laminectomy was performed at T12, and from this level an intrathecal catheter was inserted in the same operation, or 4 days, 2 weeks, or 8 weeks after the SCI. To determine the therapeutic time window for the intrathecal rhHGF, 200 µg of rhHGF was administered for 2 weeks starting immediately after the SCI; or 8, 40, or 200 µg of rhHGF was administered for 2 weeks starting 4 days after SCI; or 400 µg of rhHGF was administered for 4 weeks starting 2 or 8 weeks after SCI. These dosages were determined from previous experiments, in which rhHGF was intrathecally administered into ALS rats [29]. The contusive SCI resulted in complete paralysis, and significant motor recovery of the hindlimbs was seen when 200 µg of intrathecal rhHGF was administered for 2 weeks starting immediately after the SCI, compared to the control group treated with dilution medium. In addition, 8, 40, or 200 µg of intrathecal rhHGF administered for 2 weeks, starting 4 days after the SCI, also promoted significant functional recovery. On the other hand, functional recovery was not promoted when 400 µg of intrathecal rhHGF was administered for 4 weeks, starting 2 or 8 weeks after SCI [71]. Nishimura et al. used microarray analysis and mRNA deep sequencing to study the changes in gene expression over time after SCI in marmosets. They found that the inflammatory response in marmosets after the SCI lasted significantly longer than that in rodents; while the acute inflammatory phase resolves within one week after SCI in rodents, it may continue until 2 weeks after SCI in marmosets [77]. These findings suggest that the therapeutic time window for treating acute SCI using neurotrophic factors might be longer for non-human primates than for rodents, and support the possibility that although the therapeutic time window for intrathecal rhHGF has not been examined in marmosets, a delayed administration of intrathecal rhHGF starting 4 days after SCI could also be effective in primates.

#### 2.2.2. Minimal Effective Dose of Intrathecal rhHGF

It was noteworthy that even low doses (8 µg and 40 µg) of intrathecal rhHGF starting 4 days after SCI and administered for 2 weeks (i.e., until the end of the acute inflammatory phase [77]), also significantly promoted functional recovery [71]. Quantitative analyses using enzyme-linked immunosorbent assays (ELISAs) showed that the amount of endogenous rat HGF in the injured spinal cord was upregulated by intrathecal rhHGF administration, reaching a concentration that was 2.4, 10.7, or 15.1 times that of the control group at 10 days after SCI, when 8, 40, or 200 µg of rhHGF, respectively, was administered for 2 weeks, starting 4 days after SCI (unpublished data). These results suggested that a moderate increase in the endogenous HGF, to 2 to 10 times the untreated level, can still exert therapeutic effects. This finding was consistent with our previous report showing that upregulating the HGF level by 3–4 times promotes the recovery of function in a rat SCI model [31]. In addition to these results, we referred to pharmacokinetic studies of intrathecal rhHGF performed in cynomolgus monkeys (data not shown), as well as in a human phase I clinical trial of intrathecal rhHGF for ALS [30] to finalize the dose of intrathecal rhHGF used in the clinical trial for SCI.

#### 2.2.3. Advantages and Disadvantages of Intrathecal Administration

Clinical trials for various drugs have recently been carried out for acute SCI, in which the drug administration methods used have depended on the therapeutic mechanisms and pharmacokinetics of the drugs: granulocyte colony-stimulating factor (G-CSF) was administered intravenously [78,79,80], riluzole orally (identified as NCT00876889 and NCT01597518 in ClinicalTrials.gov) [81,82], magnesium chloride in polyethylene glycol intravenously (identified as NCT01750684 in ClinicalTrials.gov), recombinant basic fibroblast growth factor (bFGF) intravenously (identified as NCT01502631 in ClinicalTrials.gov), Rho protein antagonist by an extradural fibrin-mediated delivery system (identified as NCT00500812 and NCT02053883 in ClinicalTrials.gov) [83], ibuprofen orally (identified as NCT02096913 in ClinicalTrials.gov) [84], minocycline intravenously (identified as NCT00559494 and NCT01813240 in ClinicalTrials.gov) [85], and atorvastatin orally [86]. Oral or intravenous administration is clearly easier and less invasive than intrathecal administration. However, in applying rhHGF to human SCI patients, we think that intrathecal administration would be the best route. Although HGF can actually pass through the blood-brain barrier (BBB) [87], the BBB still limits the amount of rhHGF that can travel from the blood to spinal cord, and makes it difficult to control and maintain the concentration of intrathecal rhHGF when administered intravenously. Yamane et al. reported that it is important to maintain the concentration and location of HGF in the injured spinal cord [66], and our previous pharmacokinetic studies in cynomolgus monkeys and in a human phase I clinical trial for ALS reveal that continual intrathecal injections can directly control and maintain the therapeutic dose of intrathecal rhHGF [30]. Despite the invasiveness of intrathecal administration, these pharmacokinetic studies, as well as previous findings that appropriately administered intrathecal rhHGF can exert therapeutic effects in ALS and SCI model animals [29,70,71], strongly argue for this protocol. Finally, another reason for using intrathecal administration is that HGF is known to promote the mobility of cells that are involved in invasion–metastasis and the resistance to anticancer drugs in cancer tissues [19], and the intravenous administration of rhHGF can increase this risk.

## 3. Phase I/II Clinical Trial for Acute Cervical SCI in Japan

With these basic studies as a foundation, we conducted phase I/II clinical trials for acute cervical SCI from June 2014 to May 2018 at three major SCI centers in Japan (Hokkaido Spinal Cord Injury Center in Hokkaido, Spinal Injuries Center in Fukuoka, and Murayama Medical Center in Tokyo) (clinicaltrials.gov, registration number NCT02193334).

This trial was randomized, double-blinded, and placebo-controlled. We planned to enroll cervical SCI patients who showed modified Frankel A, B1, or B2 72 h after injury onset. Then rhHGF (KP-100IT) was intrathecally injected at the lumbar level and repeated weekly for a total of 5 times, with the first injection given within 6 h after the 72-h time point after injury onset. All patients were followed up until 24 weeks after the primary injection. At present, adverse events including the production of autoantibodies against rhHGF, and changes in the American Spinal Injury Association (ASIA) motor score from the baseline at the 72-h post-injury time point were evaluated as primary end points.

## 4. Conclusions

We revealed the changes in endogenous HGF in the rat injured spinal cord over time and the therapeutic efficacy of administering an HGF-expressing viral vector into an injured rat spinal cord. Then, from the original experimental strategy using the HSV-1 vector in rats, we developed a clinical strategy using rhHGF. We are analyzing the results of this trial at the time of writing this manuscript, November 2018, and believe that promising results will be disclosed in the near future.

## Figures and Tables

**Table 1:** Differences in the hepatocyte growth factor (HGF) supply system after tissue injury between epithelial organs and the spinal cord. Compared to epithelial organs, including the liver, lung, and kidney, the injured spinal cord has a poor ability to upregulate endogenous HGF, nor does it receive HGF supplied from other organs.

Epithelial Organs (Liver, Lung, or Kidney)	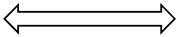	Spinal Cord
Marked increase within 24 h after injury	Endogenous upregulation of HGF in the injured organ	Weak and delayed, with a peak around 4 weeks after injury
Marked increase within 24 h after injury	Delivery of HGF from other intact organs via an endocrine mechanism	No delivery

**Table 2:** Therapeutic mechanisms of HGF on the injured spinal cord, determined using different therapeutic interventions in different types of SCI. SCI: spinal cord injury; HSV-1: herpes simplex virus-1; MSCs: mesenchymal stem cells; CSPG: chondroitin sulfate proteoglycans; CBD-HGF: engineered HGF fused with a collagen-binding domain; NSCs: neural stem cells; CST: corticospinal tract; rhHGF: recombinant human HGF.

Reference	SCI Model	Therapeutic Intervention	Timing of the Intervention	Therapeutic Effects
Kitamura et al. (2007) [31]	Contusive thoracic SCI in adult rats	HSV-1 vector injection into spinal cord	3 days prior to SCI	Promoted the survival of neurons and oligodendrocytes, angiogenesis, and the axonal regrowth of 5-HT-positive fibers
Jeong et al. (2012) [63]	Hemisectional cervical SCI in adult rats	Transplantation of HGF-overexpressing MSCs into hemisectional lesion	Immediately after SCI	Diminished the TGF isoform levels, reduced astrocyte activation, and decreased the CSPG deposition around the lesion site to increase axonal growth beyond the glial scar.
Yamane et al. (2018) [66]	Compressive thoracic SCI in adult mice	Single intrathecal injection of engineered CBD-HGF	Immediately after SCI	CBD-HGF remained in the spinal cord for 7 days and exerted an anti-inflammatory effect by disrupting NF-κB signaling, decreasing cytokine levels, and reducing the infiltration of leukocytes and glial scar formation.
Takano et al. (2017) [67]	Contusive thoracic SCI in aged and young mice	Transplantation of NSCs	9 days after SCI	HGF was the most highly expressed neurotrophic factor in aged mice compared to young ones at the time of transplantation, and promoted the survival, neuronal differentiation, and synapse formation of grafted NSCs.
Kitamura et al. (2011) [70]	Contusive cervical SCI in adult common marmosets	Intrathecal infusion of 400 µg of rhHGF for 4 weeks	Starting immediately after SCI	Preserved myelinated white matter and the CST pathway and promoted hand function
Kitamura et al. (2016) [71]	Contusive thoracic SCI in adult rats	Intrathecal infusion of rhHGF (1) 200 µg for 2 weeks, starting immediately after SCI; (2) 8, 40, or 200 µg for 2 weeks starting 4 days after SCI; (3) 400 µg for 4 weeks, starting 2 or 8 weeks after SCI		Promoted functional recovery when intrathecal infusion was started immediately after or 4 days after SCI
	More severe contusive cervical SCI than in [69] in adult common marmosets	Intrathecal infusion of 400 µg of rhHGF for 4 weeks	Starting immediately after SCI	All marmosets showed no recovery in upper limb motor function until 4 days after SCI. At least one key muscle in upper limb became useful in rhHGF-treated animals, whereas all the key muscles remained useless in the control animals.

## References

[1] Jain N.B., Ayers G.D., Peterson E.N., Harris M.B., Morse L., O’Connor K.C., Garshick E. (2015). Traumatic spinal cord injury in the United States, 1993–2012. JAMA.

[2] Sekhon L.H., Fehlings M.G. (2001). Epidemiology, demographics, and pathophysiology of acute spinal cord injury. Spine.

[3] Ho C.H., Wuermser L.A., Priebe M.M., Chiodo A.E., Scelza W.M., Kirshblum S.C. (2007). Spinal cord injury medicine. 1. Epidemiology and classification. Arch. Phys. Med. Rehabil..

[4] Kim Y.H., Ha K.Y., Kim S.I. (2017). Spinal Cord Injury and Related Clinical Trials. Clin. Orthop. Surg..

[5] Pickett G.E., Campos-Benitez M., Keller J.L., Duggal N. (2006). Epidemiology of traumatic spinal cord injury in Canada. Spine.

[6] Van den Berg M.E., Castellote J.M., Mahillo-Fernandez I., de Pedro-Cuesta J. (2010). Incidence of spinal cord injury worldwide: A systematic review. Neuroepidemiology.

[7] Oyinbo C.A. (2011). Secondary injury mechanisms in traumatic spinal cord injury: A nugget of this multiply cascade. Acta Neurobiol. Exp. (Wars).

[8] Witiw C.D., Fehlings M.G. (2015). Acute Spinal Cord Injury. J. Spinal Disord. Tech..

[9] Bracken M.B., Shepard M.J., Collins W.F., Holford T.R., Young W., Baskin D.S., Eisenberg H.M., Flamm E., Leo-Summers L., Maroon J. (1990). A randomized, controlled trial of methylprednisolone or naloxone in the treatment of acute spinal-cord injury. Results of the Second National Acute Spinal Cord Injury Study. N. Engl. J. Med..

[10] Fehlings M.G., Wilson J.R., Cho N. (2014). Methylprednisolone for the treatment of acute spinal cord injury: Counterpoint. Neurosurgery.

[11] Hurlbert R.J. (2014). Methylprednisolone for the treatment of acute spinal cord injury: Point. Neurosurgery.

[12] Hurlbert R.J., Hadley M.N., Walters B.C., Aarabi B., Dhall S.S., Gelb D.E., Rozzelle C.J., Ryken T.C., Theodore N. (2013). Pharmacological therapy for acute spinal cord injury. Neurosurgery.

[13] Matsumoto T., Tamaki T., Kawakami M., Yoshida M., Ando M., Yamada H. (2001). Early complications of high-dose methylprednisolone sodium succinate treatment in the follow-up of acute cervical spinal cord injury. Spine.

[14] Nakamura T., Nawa K., Ichihara A. (1984). Partial purification and characterization of hepatocyte growth factor from serum of hepatectomized rats. Biochem. Biophys. Res. Commun..

[15] Miyazawa K., Tsubouchi H., Naka D., Takahashi K., Okigaki M., Arakaki N., Nakayama H., Hirono S., Sakiyama O. (1989). Molecular cloning and sequence analysis of cDNA for human hepatocyte growth factor. Biochem. Biophys. Res. Commun..

[16] Nakamura T., Nishizawa T., Hagiya M., Seki T., Shimonishi M., Sugimura A., Tashiro K., Shimizu S. (1989). Molecular cloning and expression of human hepatocyte growth factor. Nature.

[17] Bottaro D.P., Rubin J.S., Faletto D.L., Chan A.M., Kmiecik T.E., Vande Woude G.F., Aaronson S.A. (1991). Identification of the hepatocyte growth factor receptor as the c-met proto-oncogene product. Science.

[18] Kato T. (2017). Biological roles of hepatocyte growth factor-Met signaling from genetically modified animals. Biomed. Rep..

[19] Matsumoto K., Funakoshi H., Takahashi H., Sakai K. (2014). HGF-Met Pathway in Regeneration and Drug Discovery. Biomedicines.

[20] Date I., Takagi N., Takagi K., Kago T., Matsumoto K., Nakamura T., Takeo S. (2004). Hepatocyte growth factor attenuates cerebral ischemia-induced learning dysfunction. Biochem. Biophys. Res. Commun..

[21] Date I., Takagi N., Takagi K., Kago T., Matsumoto K., Nakamura T., Takeo S. (2004). Hepatocyte growth factor improved learning and memory dysfunction of microsphere-embolized rats. J. Neurosci. Res..

[22] Shang J., Deguchi K., Ohta Y., Liu N., Zhang X., Tian F., Yamashita T., Ikeda Y., Matsuura T., Funakoshi H. (2011). Strong neurogenesis, angiogenesis, synaptogenesis, and antifibrosis of hepatocyte growth factor in rats brain after transient middle cerebral artery occlusion. J. Neurosci. Res..

[23] Miyazawa T., Matsumoto K., Ohmichi H., Katoh H., Yamashima T., Nakamura T. (1998). Protection of hippocampal neurons from ischemia-induced delayed neuronal death by hepatocyte growth factor: A novel neurotrophic factor. J. Cereb. Blood Flow Metab..

[24] Shimamura M., Sato N., Oshima K., Aoki M., Kurinami H., Waguri S., Uchiyama Y., Ogihara T., Kaneda Y., Morishita R. (2004). Novel therapeutic strategy to treat brain ischemia: Overexpression of hepatocyte growth factor gene reduced ischemic injury without cerebral edema in rat model. Circulation.

[25] Shimamura M., Sato N., Waguri S., Uchiyama Y., Hayashi T., Iida H., Nakamura T., Ogihara T., Kaneda Y., Morishita R. (2006). Gene transfer of hepatocyte growth factor gene improves learning and memory in the chronic stage of cerebral infarction. Hypertension.

[26] Niimura M., Takagi N., Takagi K., Funakoshi H., Nakamura T., Takeo S. (2006). Effects of hepatocyte growth factor on phosphorylation of extracellular signal-regulated kinase and hippocampal cell death in rats with transient forebrain ischemia. Eur. J. Pharmacol..

[27] Niimura M., Takagi N., Takagi K., Mizutani R., Ishihara N., Matsumoto K., Funakoshi H., Nakamura T., Takeo S. (2006). Prevention of apoptosis-inducing factor translocation is a possible mechanism for protective effects of hepatocyte growth factor against neuronal cell death in the hippocampus after transient forebrain ischemia. J. Cereb. Blood Flow Metab..

[28] Sun W., Funakoshi H., Nakamura T. (2002). Overexpression of HGF retards disease progression and prolongs life span in a transgenic mouse model of ALS. J. Neurosci..

[29] Ishigaki A., Aoki M., Nagai M., Warita H., Kato S., Kato M., Nakamura T., Funakoshi H., Itoyama Y. (2007). Intrathecal delivery of hepatocyte growth factor from amyotrophic lateral sclerosis onset suppresses disease progression in rat amyotrophic lateral sclerosis model. J. Neuropathol. Exp. Neurol..

[30] Warita H., Kato M., Asada R., Yamashita A., Hayata D., Adachi K., Aoki M. (2018). Safety, Tolerability, and Pharmacodynamics of Intrathecal Injection of Recombinant Human HGF (KP-100) in Subjects With Amyotrophic Lateral Sclerosis: A Phase I Trial. J. Clin. Pharmacol..

[31] Kitamura K., Iwanami A., Nakamura M., Yamane J., Watanabe K., Suzuki Y., Miyazawa D., Shibata S., Funakoshi H., Miyatake S. (2007). Hepatocyte growth factor promotes endogenous repair and functional recovery after spinal cord injury. J. Neurosci. Res..

[32] Kono S., Nagaike M., Matsumoto K., Nakamura T. (1992). Marked induction of hepatocyte growth factor mRNA in intact kidney and spleen in response to injury of distant organs. Biochem. Biophys. Res. Commun..

[33] Matsumoto K., Nakamura T. (1997). Hepatocyte growth factor (HGF) as a tissue organizer for organogenesis and regeneration. Biochem. Biophys. Res. Commun..

[34] Noji S., Tashiro K., Koyama E., Nohno T., Ohyama K., Taniguchi S., Nakamura T. (1990). Expression of hepatocyte growth factor gene in endothelial and Kupffer cells of damaged rat livers, as revealed by in situ hybridization. Biochem. Biophys. Res. Commun..

[35] Yanagita K., Matsumoto K., Sekiguchi K., Ishibashi H., Niho Y., Nakamura T. (1993). Hepatocyte growth factor may act as a pulmotrophic factor on lung regeneration after acute lung injury. J. Biol. Chem..

[36] Igawa T., Matsumoto K., Kanda S., Saito Y., Nakamura T. (1993). Hepatocyte growth factor may function as a renotropic factor for regeneration in rats with acute renal injury. Am. J. Physiol..

[37] Nagayama T., Nagayama M., Kohara S., Kamiguchi H., Shibuya M., Katoh Y., Itoh J., Shinohara Y. (2004). Post-ischemic delayed expression of hepatocyte growth factor and c-Met in mouse brain following focal cerebral ischemia. Brain Res..

[38] Caton A., Hacker A., Naeem A., Livet J., Maina F., Bladt F., Klein R., Birchmeier C., Guthrie S. (2000). The branchial arches and HGF are growth-promoting and chemoattractant for cranial motor axons. Development.

[39] Maina F., Klein R. (1999). Hepatocyte growth factor, a versatile signal for developing neurons. Nat. Neurosci..

[40] Hamanoue M., Takemoto N., Matsumoto K., Nakamura T., Nakajima K., Kohsaka S. (1996). Neurotrophic effect of hepatocyte growth factor on central nervous system neurons in vitro. J. Neurosci. Res..

[41] Okura Y., Arimoto H., Tanuma N., Matsumoto K., Nakamura T., Yamashima T., Miyazawa T., Matsumoto Y. (1999). Analysis of neurotrophic effects of hepatocyte growth factor in the adult hypoglossal nerve axotomy model. Eur. J. Neurosci..

[42] Hayashi K., Morishita R., Nakagami H., Yoshimura S., Hara A., Matsumoto K., Nakamura T., Ogihara T., Kaneda Y., Sakai N. (2001). Gene therapy for preventing neuronal death using hepatocyte growth factor: In vivo gene transfer of HGF to subarachnoid space prevents delayed neuronal death in gerbil hippocampal CA1 neurons. Gene Ther..

[43] Nakamura Y., Morishita R., Higaki J., Kida I., Aoki M., Moriguchi A., Yamada K., Hayashi S., Yo Y., Matsumoto K. (1995). Expression of local hepatocyte growth factor system in vascular tissues. Biochem. Biophys. Res. Commun..

[44] Nakamura Y., Morishita R., Nakamura S., Aoki M., Moriguchi A., Matsumoto K., Nakamura T., Higaki J., Ogihara T. (1996). A vascular modulator, hepatocyte growth factor, is associated with systolic pressure. Hypertension.

[45] Morishita R., Aoki M., Hashiya N., Yamasaki K., Kurinami H., Shimizu S., Makino H., Takesya Y., Azuma J., Ogihara T. (2004). Therapeutic angiogenesis using hepatocyte growth factor (HGF). Curr. Gene Ther..

[46] Loy D.N., Crawford C.H., Darnall J.B., Burke D.A., Onifer S.M., Whittemore S.R. (2002). Temporal progression of angiogenesis and basal lamina deposition after contusive spinal cord injury in the adult rat. J. Comp. Neurol..

[47] Hagg T., Oudega M. (2006). Degenerative and spontaneous regenerative processes after spinal cord injury. J. Neurotrauma.

[48] Beattie M.S., Bresnahan J.C., Komon J., Tovar C.A., Van Meter M., Anderson D.K., Faden A.I., Hsu C.Y., Noble L.J., Salzman S. (1997). Endogenous repair after spinal cord contusion injuries in the rat. Exp. Neurol..

[49] Casella G.T., Marcillo A., Bunge M.B., Wood P.M. (2002). New vascular tissue rapidly replaces neural parenchyma and vessels destroyed by a contusion injury to the rat spinal cord. Exp. Neurol..

[50] Guizar-Sahagun G., Ibarra A., Espitia A., Martinez A., Madrazo I., Franco-Bourland R.E. (2005). Glutathione monoethyl ester improves functional recovery, enhances neuron survival, and stabilizes spinal cord blood flow after spinal cord injury in rats. Neuroscience.

[51] Kawabe J., Koda M., Hashimoto M., Fujiyoshi T., Furuya T., Endo T., Okawa A., Yamazaki M. (2011). Neuroprotective effects of granulocyte colony-stimulating factor and relationship to promotion of angiogenesis after spinal cord injury in rats: Laboratory investigation. J. Neurosurg. Spine.

[52] Ebens A., Brose K., Leonardo E.D., Hanson M.G., Bladt F., Birchmeier C., Barres B.A., Tessier-Lavigne M. (1996). Hepatocyte growth factor/scatter factor is an axonal chemoattractant and a neurotrophic factor for spinal motor neurons. Neuron.

[53] Wong V., Glass D.J., Arriaga R., Yancopoulos G.D., Lindsay R.M., Conn G. (1997). Hepatocyte growth factor promotes motor neuron survival and synergizes with ciliary neurotrophic factor. J. Biol. Chem..

[54] Yamagata T., Muroya K., Mukasa T., Igarashi H., Momoi M., Tsukahara T., Arahata K., Kumagai H., Momoi T. (1995). Hepatocyte growth factor specifically expressed in microglia activated Ras in the neurons, similar to the action of neurotrophic factors. Biochem. Biophys. Res. Commun..

[55] Bregman B.S. (1987). Spinal cord transplants permit the growth of serotonergic axons across the site of neonatal spinal cord transection. Brain Res..

[56] Kim J.E., Liu B.P., Park J.H., Strittmatter S.M. (2004). Nogo-66 receptor prevents raphespinal and rubrospinal axon regeneration and limits functional recovery from spinal cord injury. Neuron.

[57] Saruhashi Y., Young W., Perkins R. (1996). The recovery of 5-HT immunoreactivity in lumbosacral spinal cord and locomotor function after thoracic hemisection. Exp. Neurol..

[58] Ikegami T., Nakamura M., Yamane J., Katoh H., Okada S., Iwanami A., Watanabe K., Ishii K., Kato F., Fujita H. (2005). Chondroitinase ABC combined with neural stem/progenitor cell transplantation enhances graft cell migration and outgrowth of growth-associated protein-43-positive fibers after rat spinal cord injury. Eur. J. Neurosci..

[59] Kobayashi N.R., Fan D.P., Giehl K.M., Bedard A.M., Wiegand S.J., Tetzlaff W. (1997). BDNF and NT-4/5 prevent atrophy of rat rubrospinal neurons after cervical axotomy, stimulate GAP-43 and Talpha1-tubulin mRNA expression, and promote axonal regeneration. J. Neurosci..

[60] Ramon-Cueto A., Plant G.W., Avila J., Bunge M.B. (1998). Long-distance axonal regeneration in the transected adult rat spinal cord is promoted by olfactory ensheathing glia transplants. J. Neurosci..

[61] Morgenstern D.A., Asher R.A., Fawcett J.W. (2002). Chondroitin sulphate proteoglycans in the CNS injury response. Prog. Brain Res..

[62] Silver J., Miller J.H. (2004). Regeneration beyond the glial scar. Nat. Rev. Neurosci..

[63] Jeong S.R., Kwon M.J., Lee H.G., Joe E.H., Lee J.H., Kim S.S., Suh-Kim H., Kim B.G. (2012). Hepatocyte growth factor reduces astrocytic scar formation and promotes axonal growth beyond glial scars after spinal cord injury. Exp. Neurol..

[64] Giannopoulou M., Dai C., Tan X., Wen X., Michalopoulos G.K., Liu Y. (2008). Hepatocyte growth factor exerts its anti-inflammatory action by disrupting nuclear factor-kappaB signaling. Am. J. Pathol..

[65] Gong R. (2008). Multi-target anti-inflammatory action of hepatocyte growth factor. Curr. Opin. Investig. Drugs.

[66] Yamane K., Mazaki T., Shiozaki Y., Yoshida A., Shinohara K., Nakamura M., Yoshida Y., Zhou D., Kitajima T., Tanaka M. (2018). Collagen-Binding Hepatocyte Growth Factor (HGF) alone or with a Gelatin- furfurylamine Hydrogel Enhances Functional Recovery in Mice after Spinal Cord Injury. Sci. Rep..

[67] Takano M., Kawabata S., Shibata S., Yasuda A., Nori S., Tsuji O., Nagoshi N., Iwanami A., Ebise H., Horiuchi K. (2017). Enhanced Functional Recovery from Spinal Cord Injury in Aged Mice after Stem Cell Transplantation through HGF Induction. Stem Cell Rep..

[68] Kokuzawa J., Yoshimura S., Kitajima H., Shinoda J., Kaku Y., Iwama T., Morishita R., Shimazaki T., Okano H., Kunisada T. (2003). Hepatocyte growth factor promotes proliferation and neuronal differentiation of neural stem cells from mouse embryos. Mol. Cell. Neurosci..

[69] Kato M., Yoshimura S., Kokuzawa J., Kitajima H., Kaku Y., Iwama T., Shinoda J., Kunisada T., Sakai N. (2004). Hepatocyte growth factor promotes neuronal differentiation of neural stem cells derived from embryonic stem cells. Neuroreport.

[70] Kitamura K., Fujiyoshi K., Yamane J., Toyota F., Hikishima K., Nomura T., Funakoshi H., Nakamura T., Aoki M., Toyama Y. (2011). Human hepatocyte growth factor promotes functional recovery in primates after spinal cord injury. PLoS ONE.

[71] Kitamura K., Iwanami A., Iwai H., Toyama Y., Matsumoto M., Okano H., Nakamura M. (2016). Therapeutic time window and preclinical efficacy of intrathecal administration of recombinant human hepatocyte growth factor for acute spinal cord injury. J. Spine Res..

[72] Lemon R. (2004). Cortico-motoneuronal system and dexterous finger movements. J. Neurophysiol..

[73] Yamane J., Nakamura M., Iwanami A., Sakaguchi M., Katoh H., Yamada M., Momoshima S., Miyao S., Ishii K., Tamaoki N. (2010). Transplantation of galectin-1-expressing human neural stem cells into the injured spinal cord of adult common marmosets. J. Neurosci. Res..

[74] Iwanami A., Yamane J., Katoh H., Nakamura M., Momoshima S., Ishii H., Tanioka Y., Tamaoki N., Nomura T., Toyama Y. (2005). Establishment of graded spinal cord injury model in a nonhuman primate: The common marmoset. J. Neurosci. Res..

[75] Iwanami A., Kaneko S., Nakamura M., Kanemura Y., Mori H., Kobayashi S., Yamasaki M., Momoshima S., Ishii H., Ando K. (2005). Transplantation of human neural stem cells for spinal cord injury in primates. J. Neurosci. Res..

[76] Fujiyoshi K., Yamada M., Nakamura M., Yamane J., Katoh H., Kitamura K., Kawai K., Okada S., Momoshima S., Toyama Y. (2007). In vivo tracing of neural tracts in the intact and injured spinal cord of marmosets by diffusion tensor tractography. J. Neurosci..

[77] Nishimura S., Sasaki T., Shimizu A., Yoshida K., Iwai H., Koya I., Kobayashi Y., Itakura G., Shibata S., Ebise H. (2014). Global gene expression analysis following spinal cord injury in non-human primates. Exp. Neurol..

[78] Inada T., Takahashi H., Yamazaki M., Okawa A., Sakuma T., Kato K., Hashimoto M., Hayashi K., Furuya T., Fujiyoshi T. (2014). Multicenter prospective nonrandomized controlled clinical trial to prove neurotherapeutic effects of granulocyte colony-stimulating factor for acute spinal cord injury: Analyses of follow-up cases after at least 1 year. Spine.

[79] Kamiya K., Koda M., Furuya T., Kato K., Takahashi H., Sakuma T., Inada T., Ota M., Maki S., Okawa A. (2015). Neuroprotective therapy with granulocyte colony-stimulating factor in acute spinal cord injury: A comparison with high-dose methylprednisolone as a historical control. Eur. Spine J..

[80] Koda M., Hanaoka H., Sato T., Fujii Y., Hanawa M., Takahashi S., Furuya T., Ijima Y., Saito J., Kitamura M. (2018). Study protocol for the G-SPIRIT trial: A randomised, placebo-controlled, double-blinded phase III trial of granulocyte colony-stimulating factor-mediated neuroprotection for acute spinal cord injury. BMJ Open.

[81] Fehlings M.G., Nakashima H., Nagoshi N., Chow D.S., Grossman R.G., Kopjar B. (2016). Rationale, design and critical end points for the Riluzole in Acute Spinal Cord Injury Study (RISCIS): A randomized, double-blinded, placebo-controlled parallel multi-center trial. Spinal Cord..

[82] Fehlings M.G., Wilson J.R., Frankowski R.F., Toups E.G., Aarabi B., Harrop J.S., Shaffrey C.I., Harkema S.J., Guest J.D., Tator C.H. (2012). Riluzole for the treatment of acute traumatic spinal cord injury: Rationale for and design of the NACTN Phase I clinical trial. J. Neurosurg. Spine.

[83] Fehlings M.G., Theodore N., Harrop J., Maurais G., Kuntz C., Shaffrey C.I., Kwon B.K., Chapman J., Yee A., Tighe A. (2011). A phase I/IIa clinical trial of a recombinant Rho protein antagonist in acute spinal cord injury. J. Neurotrauma.

[84] Kopp M.A., Liebscher T., Watzlawick R., Martus P., Laufer S., Blex C., Schindler R., Jungehulsing G.J., Knuppel S., Kreutztrager M. (2016). SCISSOR-Spinal Cord Injury Study on Small molecule-derived Rho inhibition: A clinical study protocol. BMJ Open.

[85] Casha S., Zygun D., McGowan M.D., Bains I., Yong V.W., Hurlbert R.J. (2012). Results of a phase II placebo-controlled randomized trial of minocycline in acute spinal cord injury. Brain.

[86] Aghazadeh J., Samadi Motlagh P., Salehpour F., Meshkini A., Fatehi M., Mirzaei F., Naseri Alavi S.A. (2017). Effects of Atorvastatin in Patients with Acute Spinal Cord Injury. Asian Spine J..

[87] Pan W., Yu Y., Yemane R., Cain C., Yu C., Kastin A.J. (2006). Permeation of hepatocyte growth factor across the blood-brain barrier. Exp. Neurol..

